# Dual effect of the Valsalva maneuver on autonomic nervous system activity, intraocular pressure, Schlemm’s canal, and iridocorneal angle morphology

**DOI:** 10.1186/s12886-019-1275-y

**Published:** 2020-01-03

**Authors:** Li Sun, Wei Chen, Zhiqi Chen, Yan Xiang, Jingmin Guo, Tian Hu, Qiongfang Xu, Hong Zhang, Junming Wang

**Affiliations:** Department of Ophthalmology, Tongji Hospital, Tongji Medical College, Huazhong University of Science and Technology, 1095 Jiefang Avenue, Wuhan, 430030 Hubei China

**Keywords:** The Valsalva maneuver, Schlemm’s canal, Intraocular pressure, Autonomic nervous system, Iridocorneal angle morphology

## Abstract

**Background:**

The Valsalva maneuver (VM) is widely used in daily life, and has been reported to cause high intraocular pressure (IOP). This study aimed to assess changes in IOP, the Schlemm’s canal (SC), autonomic nervous system activity, and iridocorneal angle morphology in healthy individuals during different phases of the VM.

**Methods:**

The high frequency (HF) of heart rate (HR) variability, the ratio of low frequency power (LF) and HF (LF/HF), heart rate (HR), IOP, systolic (SBP) and diastolic blood pressure (DBP), the area of SC (SCAR), pupil diameter (PD), and some iridocorneal angle parameters (AOD500, ARA750, TIA500 and TISA500) were measured in 29 young healthy individuals at baseline, phase 2, and phase 4 of the VM. SBP and DBP were measured to calculate mean arterial pressure (MAP) and mean ocular perfusion pressure (MOPP). HF and the LF/HF ratio were recorded using Kubios HR variability premium software to evaluate autonomic nervous system activity. The profiles of the anterior chamber were captured by a Spectralis optical coherence tomography device (anterior segment module).

**Results:**

Compared with baseline values, in phase 2 of the VM, HR, LF/HF, IOP (15.1 ± 2.7 vs. 18.8 ± 3.5 mmHg, *P* < 0.001), SCAR (mean) (7712.112 ± 2992.14 vs. 8921.12 ± 4482.79 μm^2^, *P* = 0.039), and PD increased significantly, whereas MOPP, AOD500, TIA500, and TISA500 decreased significantly. In phase 4, DBP, MAP, AOD500, ARA750, TIA500and TISA500 were significantly lower than baseline value, while PD and HF were remarkably larger than baseline. The comparison between phase 2 and phase 4 showed that HR, IOP (18.8 ± 3.5 vs. 14.7 ± 2.9 mmHg, *P* < 0.001) and PD decreased significantly from phase 2 to phase 4, but there were no significant differences in other parameters.

**Conclusions:**

The expansion and collapse of the SC in different phases of the VM may arise from changes in autonomic nervous system activity. Further, the effects of the VM on IOP may be attributed to changes in blood flow and ocular anatomy.

**Trial registration:**

This observational study was approved by the ethics committee of Tongji Hospital (Registration Number: ChiCTR-OON-16007850, Date: 01.28.2016).

## Background

The original Valsalva maneuver (VM) was first described by Mario Antonio Valsalva [1], and the research method was then standardized by Levin, where subjects were asked to blow into a tube and to maintain a pressure of 40 mmHg for 10 s [2, 3]. After further evaluation of the effects of varying the parameters, Benarroch et al. suggested use of 15 s strain phase during the VM [4].

The VM is considered to consist of 4 phases. In phase 1, increasing intrathoracic pressure caused by the initial straining during the maneuver translates to the arterial circulation. In phase 2, the strain is maintained, increased intrathoracic pressure and decreased venous return cause a decrease in blood pressure, with a reflexive increased heart rate (HR) because of reduced parasympathetic and increased sympathetic nervous system activity. In phase 3, release of the strain causes a rapid drop in intrathoracic pressure leading to a transient drop of blood pressure. In phase 4, the impediment to venous return to the heart is removed, and blood is ejected into the constricted vasculature by the heart, causing a pressure overshoot. Finally, parasympathetic activity is reflexively increased, resulting in a relatively quick slowing down of the heart [5–8].

There is also evidence that the VM may lead to an increase in intraocular pressure (IOP), although the mechanisms remains unclear [3, 9–11]. Schuman et al. suggested that an elevation in IOP is caused by increased uveal volume via the VM, although the observed IOP was much less than the calculated effect on IOP based on the measured change in uveal volume [12]. Alternatively, Raczynski et al. reported that an increase in IOP was related to an increase in electromyographic activity during the VM [13]. By contrast, Stuart et al. found no association of IOP changes with the electromyographic increase during the VM, but rather an influence of the autonomic nervous system [5]. We previously reported that a decrease in IOP was associated with sympathetic nerve stimulation during aerobic exercise [14], while parasympathetic stimulation caused by the water-drinking test may cause collapse of Schlemm’s canal (SC) and an increase in IOP [15].

The VM is commonly performed during daily life, and is also a diagnostic technique in clinical practice [1, 7]. Elevation and fluctuation of IOP are associated with development and progression of glaucoma [16]. Thus, in the present study, we examined the autonomic influence on IOP fluctuations and other ocular parameters in different phases of the VM.

## Methods

### Subjects

A total of 29 healthy individuals were recruited from students at Tongji Medical College, Huazhong University of Science and Technology. All participants signed written informed consent before entering the study, and the study was conducted in accordance with the tenets of the Declaration of Helsinki. All subjects underwent an ophthalmic examination, and data from their right eyes were included in the study.

The criteria for inclusion of subjects were: (1) at least 18 years old; (2) IOP of 10–21 mmHg; (3) with a normal anterior chamber depth and open angle. Further, subjects should not have ingested caffeine for at least 24 h before the studies started, and should have no history of receiving any medicines affecting the circulatory system within 1 month prior to evaluation.

Exclusion criteria were as follows: (1) systemic diseases (e.g., hypertension, diabetes, and severe cardiopulmonary insufficiency), or a family history of these conditions; (2) current ocular diseases or previous ocular surgery; (3) refractive error (RE) ≤ − 6.0 D and RE ≥3.0 D, or best corrected visual acuity < 0.5 (to ensure that the subjects had good central fixation); (4) best corrected visual acuity (BCVA) < 0.5; (5) abnormal pupil reflexes; and (6) poor compliance in performing VM correctly.

Every subject struck a correct sitting pose on the measuring instrument before blowing, and held positions during the whole VM, after which the measurements were taken. All examinations were performed following standard operating procedures. In this study, no contact was required to avoid the influence of corneal contact on the parameters and participants’ health.

### Standardized Valsalva maneuver

Every subject was trained to perform a standardized VM. Subjects were asked to exhale into a mouthpiece connected to a mercury manometer, and to maintain an expiratory pressure of 40 mmHg for approximately 15 s to complete the image acquisition process. After training, every individual was able to manage the maneuver well. The resting state before breath holding, the continuous blowing state, and the immediate recovery of normal breathing state were recorded as baseline, phase 2, and phase 4, respectively of the VM. Each phase took 15 s. Participants were given a short break of at least 5 min between every 2 VMs. Each individual performed the maneuver a total of 5 times.

### Measurement of blood pressure, HR, and electrocardiograms

The systolic blood pressure (SBP) and diastolic blood pressure (DBP) at baseline, phase 2, and phase 4 of the VM for each individual were measured using an automatic sphygmomanometer (OmronHEM-7201; Omron, Dalian, Liaoning, China). The mean arterial pressure (MAP) was calculated by the equation: MAP = DBP + (SBP - DBP)/3. Electrocardiograms were monitoring in real-time during the entire process, including in the resting state, continuous blowing state and immediately recovered normal breathing(15 s per period). HR was determined by measuring the R-R intervals. The heart rate variability (HRV) parameters of individuals were calculated in each state (baseline, phase 2, and phase 4 of the VM) by using software (Kubios HRV premium v 2.2; University of Eastern Finland).

### Anterior optical coherence tomography imaging

In a sitting position, all participants received an anterior optical coherence tomography (AS-OCT) examination (Visante OCT; Carl Zeiss Meditec, Dublin, USA.). Rectangular AS-OCT scans of the frontal, nasal, and temporal sides were collected in 3 phases. For frontal scans, the scan angle was horizontal (with nasal and temporal angles at 0°-180°) across the center of the pupil in 1 single image, while the subject stared at the internal fixation point. All AS-OCT tests were performed under standardized darkroom photopic condition (approximately 3.5 lx).

### Measurements of SC and pupil diameter

**Fig. 1:**
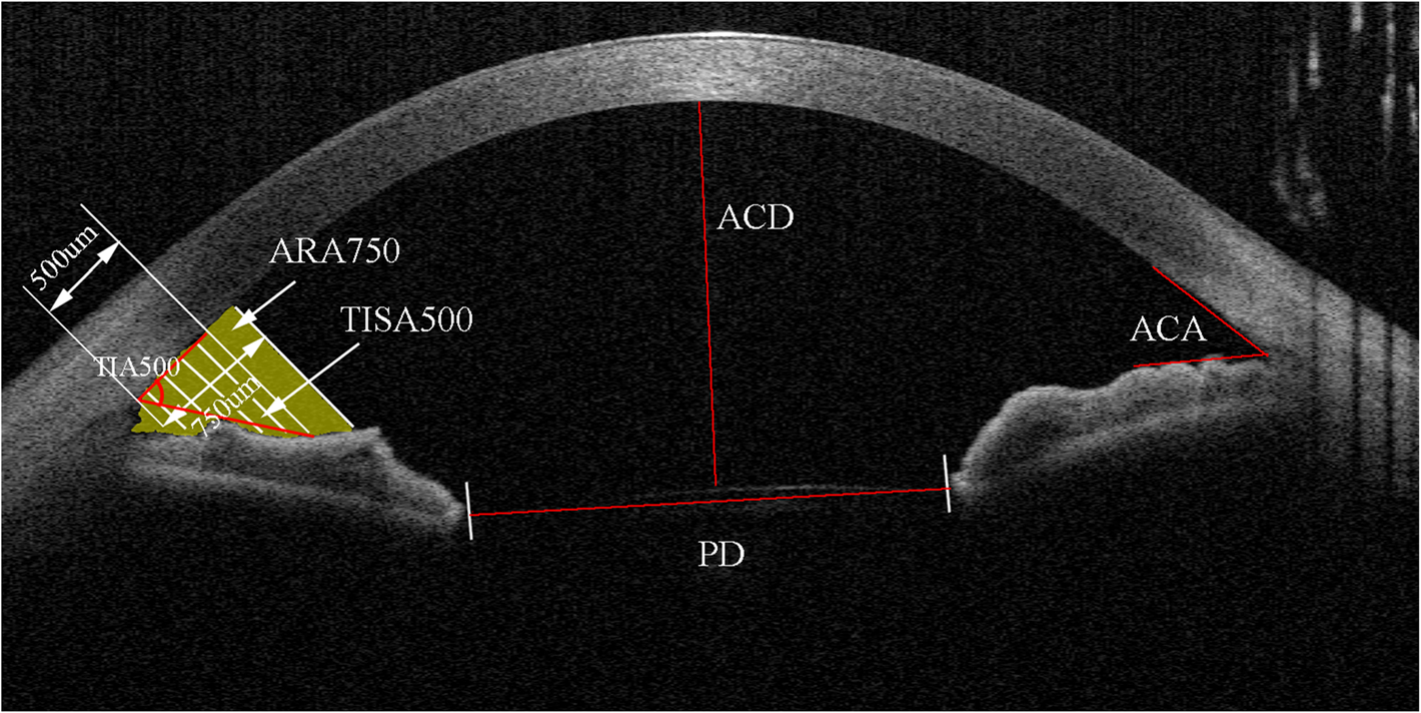
Anterior segment optical coherence tomography image. Anterior segment optical coherence tomography image showing the measurements of ACA, AOD500, ARA500, TIA500, TISA500, ACD, and pupil diameter (PD)

**Fig. 2:**
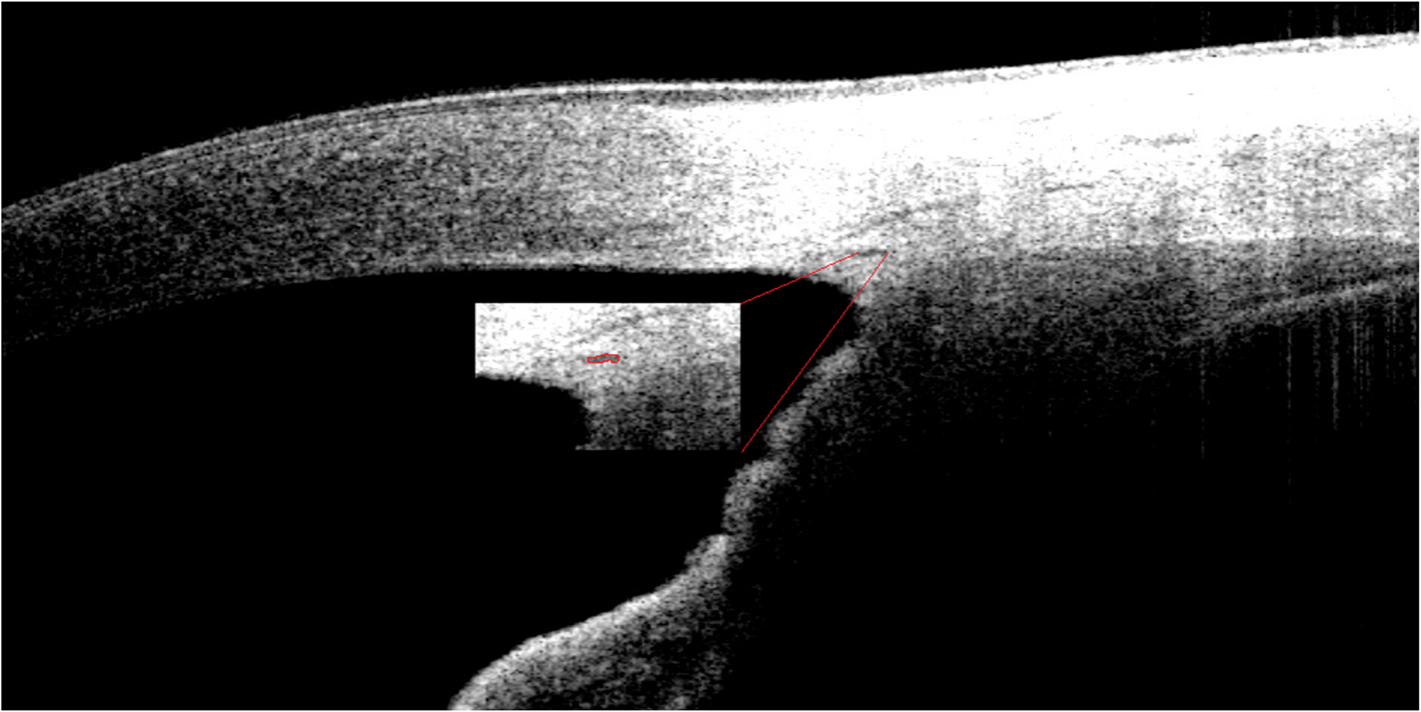
Image showing SC. The red curve indicates the SC

Anterior chamber depth (ACD), the angle opening distance at 500 μm from the scleral spur (AOD500), the angle recess area at 750 μm from the scleral spur (ARA750), trabecular iris angle at 500 μm from the scleral spur (TIA500), and trabecular-iris space area at 500 μm from the scleral spur (TISA500) were measured by the built-in 2-dimensional analysis function of the Visante OCT. ACD was defined as the length of the central perpendicular line between the posterior surface of the cornea and the anterior surface of the lens. The anterior chamber angle was defined as the arms of the posterior cornea and opposite peripheral iris, with its apex in the angle recess (Fig. 1). The SC was defined as observable when a thin, black, lucent space was detected in the images (Fig. 2). The area of the SC (SCAR; μm^2^) in the same location of the nasal and temporal sides was measured using imaging software (Image J v1.45S; National Institutes of Health, Bethesda, MD, USA). The mean SCAR was calculated as the averaged SCAR of the nasal and temporal regions. The distance from 1 side of the pupillary tip of the iris to the opposite side on images acquired by AS-OCT was measured as the pupil diameter (PD). Measurements of SCAR and PD were performed by 2 observers, and the data were recorded and stored for later statistical analysis.

### Measurement of IOP

The IOP at baseline, phase 2, and phase 4 of the VM were measured using a noncontact tonometer (NIDEK RT-2100; Nidek, Co., Ltd., Gamagori, Japan). The averaged IOP was calculated from measurements and recorded as the result. The mean ocular perfusion pressure (MOPP) was calculated as: MOPP = 2/3MAP-IOP [17].

### Statistical analysis

All statistical analyses were performed using statistical software (SPSS v 22.0; Inc., Chicago, IL, USA), and data were plotted with graphing software (GraphPad Prism v7.0; GraphPad Software, USA). The intraclass correlation coefficients test was used to analyze the re-test reliability of the measurements of SCAR and PD, which were performed by 2 observers. All applicable data are presented as the mean ± standard deviation. Repeated measures analysis of variance was used to detect differences between every 2 different phases. Univariate linear regression analysis was adopted to examine the relationship between SCAR (mean) and HF, LF/HF and IOP. All tests were 2-tailed, and statistical significance was defined as a *P* value < 0.05.

## Results

Twenty-nine individuals were enrolled in this study. A total of 29 right eyes (13 men; 16 women) were included in the analyses. Baseline and demographic characteristics are shown in Table 1. The mean patient age was 23.83 ± 3.81 years, the mean best corrected visual acuity was 1.04 ± 0.17, the mean RE was − 2.59 ± 2.48 (D), the mean ACD was 3.67 ± 0.04, and the mean body mass index was 21.16 ± 3.42. For intraclass correlation coefficient tests for measurements of SCAR and PD, the reliability coefficients were 0.85 and 0.98, respectively.

**Table 1:** Demographic and baseline characteristics of participants

Characteristics	Subjects
Number of patients (eyes)	29
Mean age, years	23.83 ± 3.81
Sex (male/female)	13/16
RE, D	−2.59 ± 2.48
BCVA	1.04 ± 0.17
BMI	21.16 ± 3.42
SBP, mmHg	118.46 ± 13.73
DBP, mmHg	77.50 ± 9.56
MAP, mmHg	91.15 ± 10.36
HR, bpm	83 ± 11.73
HF, mm^2^	1206.04 ± 1206.07
LF/HF	1.44 ± 1.64
IOP, mmHg	15.1 ± 2.7
MOPP, mmHg	46.06 ± 6.61
SCAR (mean), μm^2^	7712.112 ± 2992.14
PD, mm	4.23 ± 0.82
ACD, mm	3.67 ± 0.04
AOD500, mm	0.72 ± 0.17
ARA750, mm^2^	0.49 ± 0.12
TIA500, degree	57.51 ± 8.48
TISA500, mm^2^	0.27 ± 0.07

**Fig. 3:**
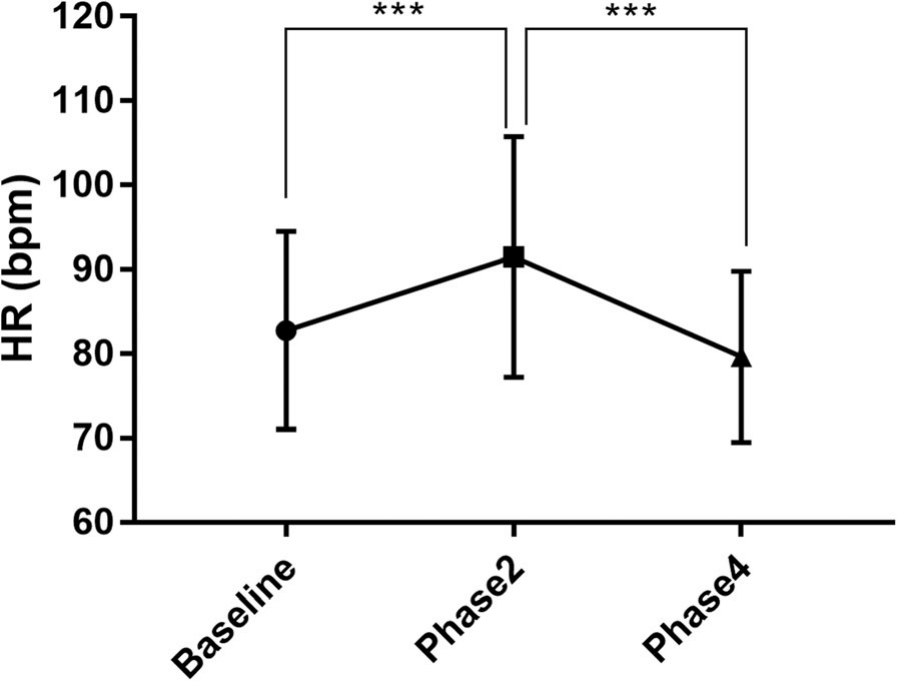
Changes in HR. HR varied significantly in phase2 and phase4 of VM compared with baseline

Table 2 shows the changes in baseline and demographic parameters during the VM. Compared with baseline, there was a significant change in BP during the phase 4 of the VM, including DBP (77.50 ± 9.56 vs. 72.63 ± 8.99 mmHg, *P* = 0.004) and MAP (91.15 ± 10.36 vs. 87.51 ± 7.32 mmHg, *P* = 0.028), while there were no changes in other BP values different states. There was also a significant increase in HR between baseline and phase 2 (83 ± 11.73 vs. 92 ± 14.28 beats/min [bpm], *P* < 0.001), and a significant decrease in HR between phase 2 and phase 4 (80 ± 10.15 bpm, *P* < 0.001) of the VM (Fig. 3). For HRV, there was a significant increase in high frequency (HF) indices at phase4 is compared with baseline (2546.08 ± 1837.11 vs. 1206.04 ± 1206.07 mm^2^, *P* = 0.007), and at phase2 compared with baseline (835.63 ± 870.92 mm2, *P* < 0.001). The ratio of low frequency power and high frequency power (LF/HF) indices also significantly increased from baseline to phase2 (1.44 ± 1.64 vs. 7.48 ± 11.61, *P* = 0.037).

**Table 2:** Changes in baseline and demographic parameters during phase 2 and phase 4 of the VM

Parameter	During phase 2 of the VM	During Phase 4 of the VM	Mean difference between baseline and phase 2	*P*_1_^b^	Mean difference between baseline and phase 4	*P*_2_^b^	Mean difference between phase 2 and phase 4	*P*3^b^
SBP, mmHg	117.79 ± 15.83	117.29 ± 11.91	0.67	1.000	1.17	1.000	0.50	1.000
DBP, mmHg	75.46 ± 11.20	72.63 ± 8.99	2.04	1.000	4.88	0.004^a^	3.83	0.649
MAP, mmHg	89.57 ± 10.83	87.51 ± 7.32	1.58	1.000	3.64	0.028^a^	2.06	0.87
HR, bpm	92 ± 14.28	80 ± 10.15	−11	0.000^a^	3	0.089	12	0.000^a^
HF, mm^2^	835.63 ± 870.92	2546.08 ± 1837.11	370.417	0.636	− 1340.042	0.007^a^	− 1710.458	0.000^a^
LF/HF	7.48 ± 11.61	5.89 ± 11.93	−6.03	0.037^a^	−4.45	0.809	1.58	1.000
IOP, mmHg	18.8 ± 3.5	14.7 ± 2.9	−3.7	0.000^a^	0.4	0.337	4.1	0.000^a^
MOPP, mmHg	41.23 ± 7.49	44.53 ± 6.15	4.83	0.005^a^	1.53	0.311	−3.30	0.051
SCAR (mean), μm^2^	8921.12 ± 4482.79	7373.08 ± 2651.92	− 1209.01	0.039^a^	339.03	1.000	1548.04	0.261
PD, mm	4.74 ± 0.74	4.53 ± 0.68	−0.52	0.000^a^	−0.31	0.000^a^	−0.21	0.050^a^
AOD500, mm	0.64 ± 0.17	0.65 ± 0.18	0.094	0.009^a^	0.079	0.008^a^	−0.015	1.000
ARA750, mm^2^	0.45 ± 0.13	0.46 ± 0.13	0.059	0.119	0.057	0.020^a^	−0.002	1.000
TIA500, °	52.57 ± 7.88	54.35 ± 9.64	6.175	0.003^a^	4.265	0.006^a^	−1.910	0.783
TISA500, mm^2^	0.24 ± 0.07	0.25 ± 0.07	0.03	0.022^a^	0.02	0.019^a^	−0.01	1.000

^a^Shows results with a significant difference

^b^Comparison using repeated measures ANOVA

*P*_1_, p value between baseline and phase 2; *P*_2_, *p* value between baseline and phase 4; *P*_3_, p value between phase 2 and phase 4

**Fig. 4:**
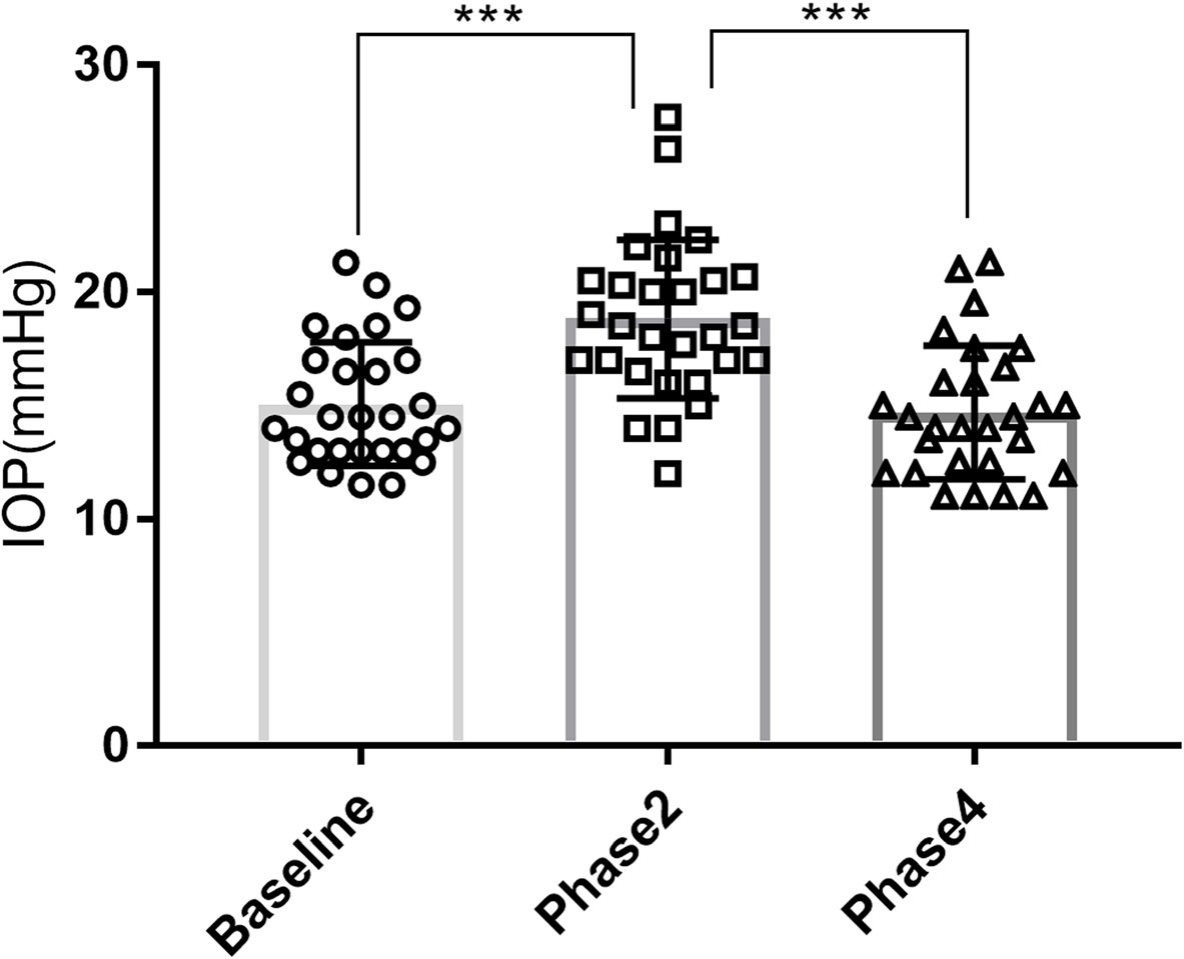
Changes in IOP. Changes in IOP in different phases during the VM

There was a significant increase in IOP from baseline to phase 2 (15.1 ± 2.7 vs. 18.8 ± 3.5 mmHg, *P* < 0.001) and a significant decrease from phase 2 to phase 4 (18.8 ± 3.5 vs. 14.7 ± 2.9 mmHg, *P* < 0.001). However, there were no differences in the IOP between baseline and phase 4 (15.1 ± 2.7 vs. 14.7 ± 2.9 mmHg, *P* = 0.337). (Fig. 4).

**Fig. 5:**
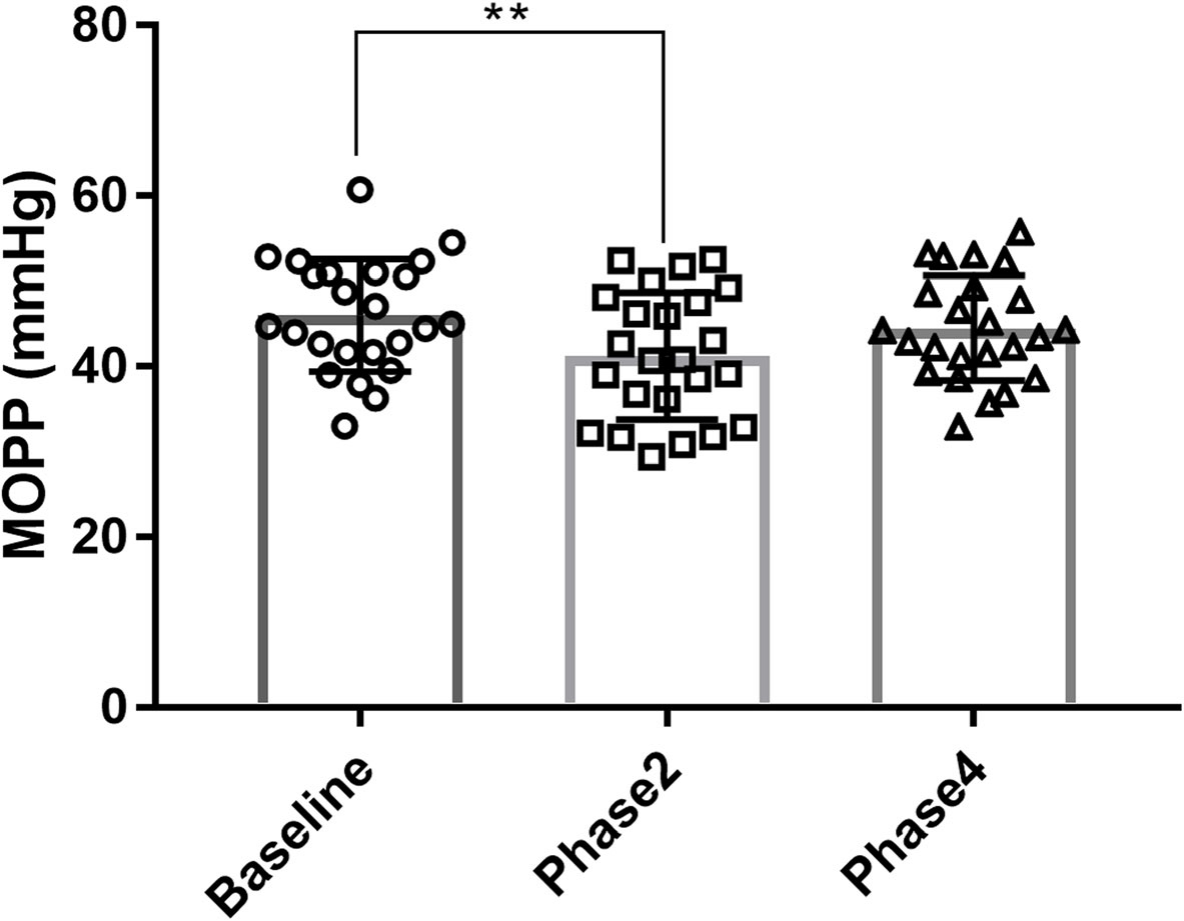
Changes in MOPP. Changes in MOPP in different phases during the VM

During phase 2 of the VM, there was a significant decrease in MOPP compared with baseline (46.06 ± 6.61 vs. 41.23 ± 7.49 mmHg, *P* = 0.005). And the increase from phase 2 to phase 4 is not significant (41.23 ± 7.49 vs. 44.53 ± 6.15 mmHg, *P* = 0.051). Further, there was no difference in MOPP between baseline and phase 4 (*P* = 0.311). (Fig. 5).

**Fig. 6:**
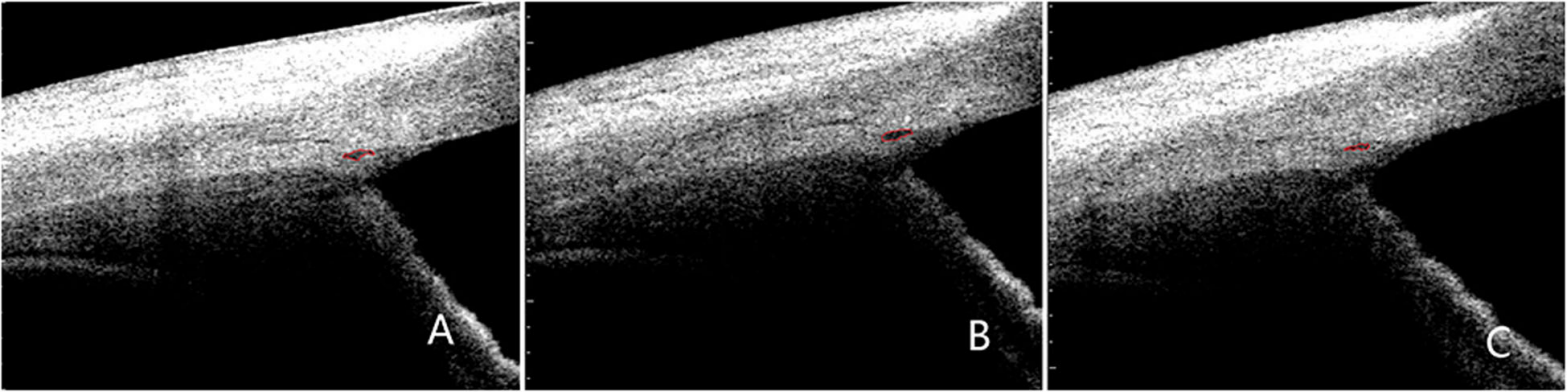
Morphology of SC (circled by red line). Baseline (A), phase 2 (B) and phase 4 (C) of the VM

**Fig. 7:**
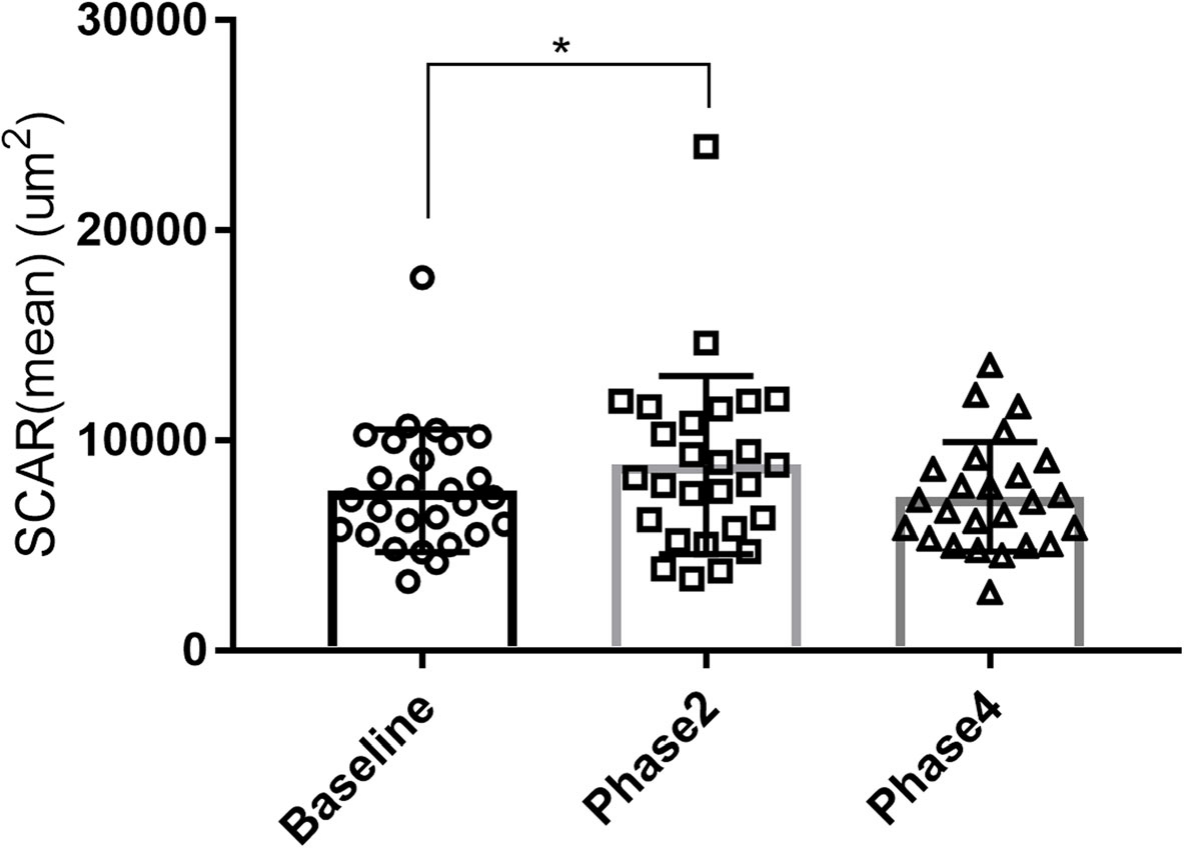
Changes in SCAR. Changes in SCAR (mean) at different phases of VM

Images of 1 eye were excluded because of low image quality. The mean SCAR increased significantly from baseline to phase 2 (7712.112 ± 2992.14 vs. 8921.12 ± 4482.79 μm2, *P* = 0.039; Table 2). The differences among other states was not significant (Fig. 6, Fig. 7).

**Fig. 8:**
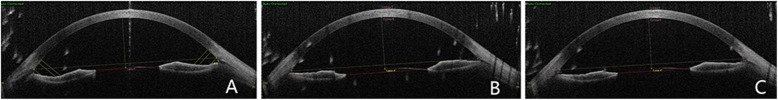
Measurement of PD (red line). Baseline (**a**), phase 2 (**b**) and phase 4 (**c**) of the VM

**Fig. 9:**
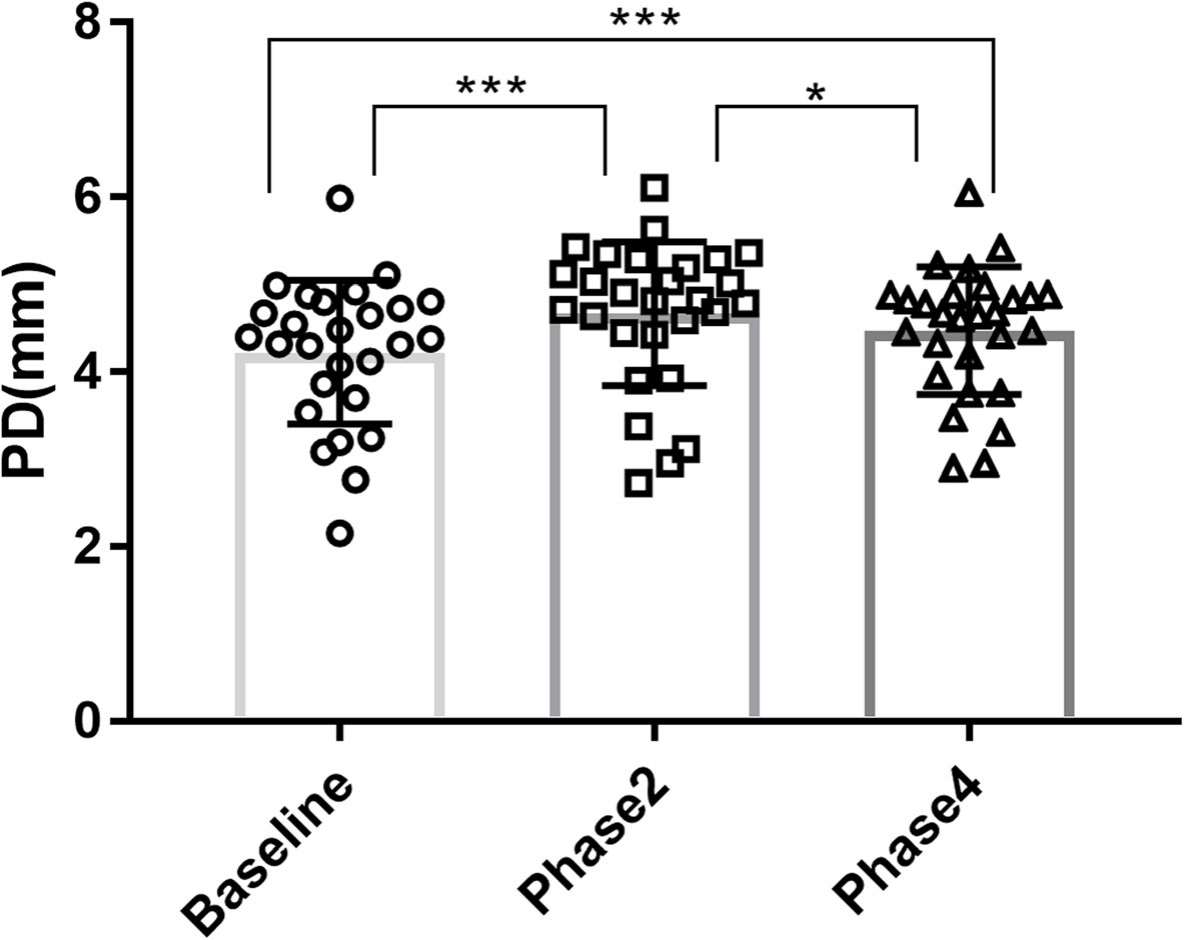
Changes in PD. Changes in PD at different phases of VM

Compared with baseline, there was a significant increase (12.1%) in PD in the phase 2 of the VM (4.23 ± 0.82 vs. 4.74 ± 0.74 mm, *P* < 0.001), and a significant decrease in PD from phase 2 to phase 4 (4.74 ± 0.74 vs. 4.53 ± 0.68 mm, *P* = 0.050). Further, there was a significant difference in PD between baseline and phase 4 (4.23 ± 0.82 vs. 4.53 ± 0.68 mm, *P* < 0.001; Fig. 8, Fig. 9).

**Fig. 10:**
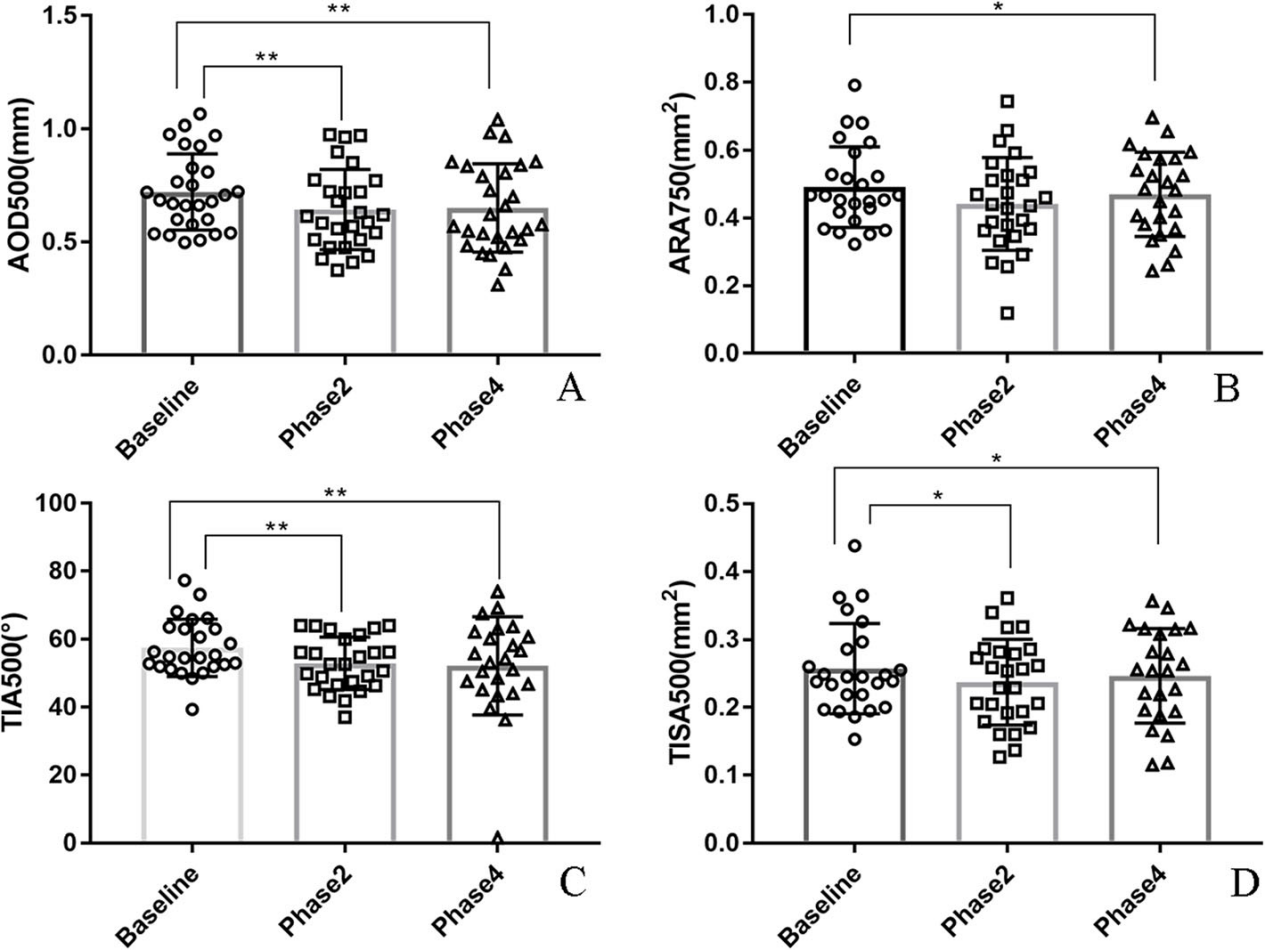
Changes in iridocorneal angle parameters. **a**-**d** Changes in AOD500, ARA750, TIA500, and TISA500 at different phases in VM

Finally, there were significant changes in AOD500, ARA750, TIA500, and TISA500 of the horizontal scan of AS-OCT during the VM. Specifically, there was a significant reduction in AOD500 (0.72 ± 0.17 vs. 0.64 ± 0.17 mm, *P* = 0.009), TIA500 (57.51 ± 8.48 vs. 52.57 ± 7.88 °, *P* = 0.003), and TISA500 (0.26 ± 0.07 vs. 0.24 ± 0.07 mm2, *P* = 0.022) from baseline to phase 2.Further, compared with baseline, the AOD500 (0.72 ± 0.17 vs. 0.65 ± 0.18 mm, *P* = 0.008), ARA750 (0.49 ± 0.12 vs. 0.46 ± 0.13 mm^2^, *P* = 0.020), TIA500 (57.51 ± 8.48 vs. 54.35 ± 9.64°, *P* = 0.003) and TISA500(0.27 ± 0.07 vs. 0.25 ± 0.07 mm^2^, *P* = 0.019) remained significantly lower in phase4 (Fig. 10 A-D).

**Fig. 11:**
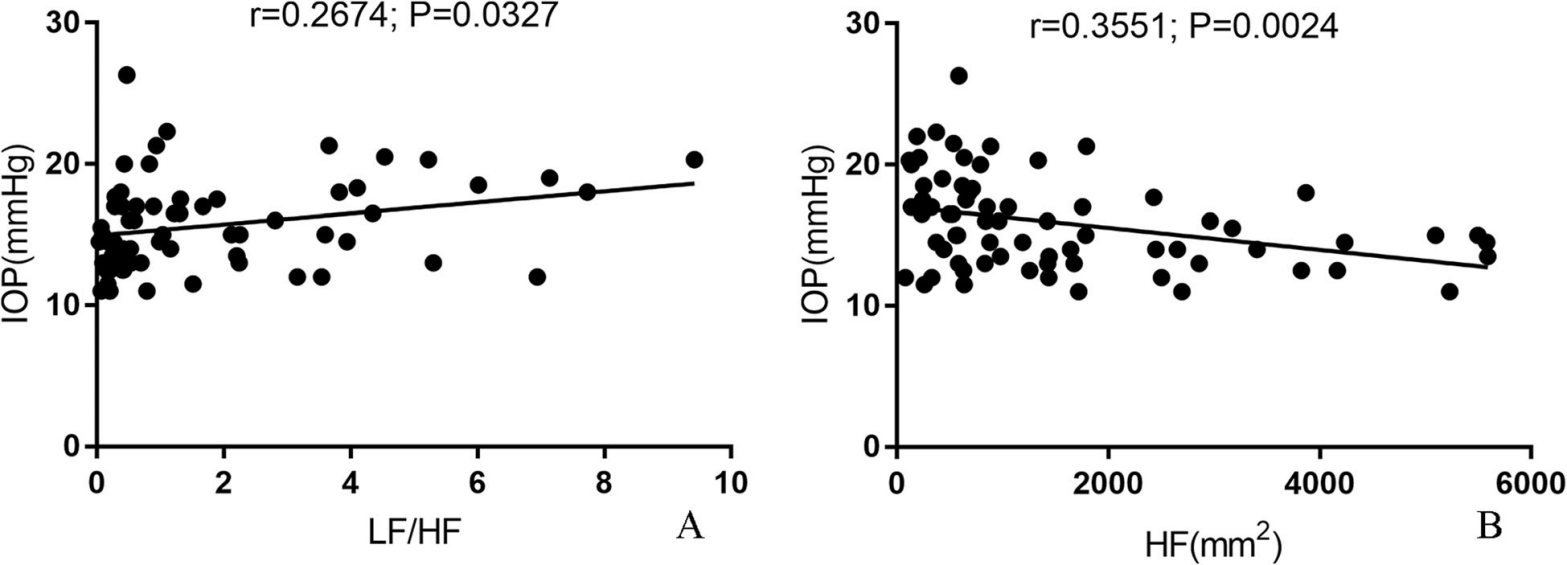
Linear correlations between LF/HF, HF and IOP. **a**, **b** The univariate regression analysis shows significant correlations of IOP with LF/HF and HF

There were also significant associations of IOP with HRV (LF/HF and HF) from baseline to maximum change (Table 3, Fig. 11a, b).

**Table 3:** Linear correlations between LF/HF, HF and IOP

Parameters	Equation	*P* value	R square
LF/HF vs. IOP	Y = 0.0234^a^X + 15.77	0.0327^a^	0.0715
HF vs. IOP	Y = − 0.0007766^a^X + 17.09	0.0024^a^	0.1261
SCAR (mean) vs. IOP	Y = −4.375e-006^a^X + 15.98	0.1618	0.0334

^a^Shows a significant linear correlation

## Discussion

The VM, is widely used to examine autonomic nervous system function, and is divided into 4 physiological phases [3, 7]. Physiological variations in phase 2 and phase 4 of the VM are accompanied by changes in both autonomic excitability and hemodynamics. The increased intrathoracic pressure causes an obstruction of venous reflux in phase 2, stimulating increased sympathetic excitability, while parasympathetic activity is increased in phase 4. As such, we selected baseline, phase 2, and phase 4 in the present study to detect the dual effects of VM on various parameters in individuals at different phases [5–7, 10]. HRV is a simple, non-invasive method to evaluate autonomic nervous system regulation, and is used in a variety of clinical situations. HRV measures the variation in the time interval between each heartbeat, which is recorded as R – R intervals [18]. Traditional HRV assessment methods include time domain, frequency domain, and nonlinear analyses. LF (0.04–0.15 Hz) and HF (0.15–0.4 Hz) are 2 of basic components of the frequency domains. A higher HF specifically indicates an increase in parasympathetic activation, while a lower LF is generally considered to be the combined sympathetic and parasympathetic influence, although this remains controversial [14, 15]. An increasing LF/HF represents a predominant sympathetic activation [19].

The key findings of the present study were the significant increase in the LF/HF ratio in phase 2 compare with baseline, reflecting an increased sympathetic activity, and the significant increase in HF indices in phase 4 compared with baseline, reflecting parasympathetic hyperfunction. Further, there was a significant increase in HR in phase 2 compared with baseline. These data are consistent with previous studies [6–8]. We also found a dilation of the PD in phase 2 compared with baseline, which then declined markedly in phase 4, although still remained higher than baseline. As the dilator pupilae is primarily controlled by sympathetic nervous system, and the sphincter pupillae is controlled by parasympathetic nervous system [20], these data suggest that activation of the autonomic nervous system caused by the VM was sufficient to invoke pupillary changes.

The elevation in IOP in healthy individuals during the continued strain of the VM has been widely reported in numerous studies [3, 9, 10, 21]. The elevation in IOP during phase 2 of the VM is thought to be predominantly caused by raised episcleral pressure, reducing aqueous outflow. The engorged anterior choroidal vessels may cause a small increase in total ocular volume, resulting in an elevation of IOP, because the wall of the eye has some rigidity [22]. Li et al. reported thickening of the anterior choroid and the ciliary body, but not of the posterior choroid, during forced exhalation against a closed airway in phase 2 [23]. We also found an elevation in IOP in phase 2 of the VM in young healthy adults compared with baseline. Significantly decreased AOD500, ARA750, TIA500 and TISA500 during the VM may also contribute to the elevated IOP, because a narrowed anterior chamber may lead to higher outflow resistance of the aqueous humor [24]. In the present study, IOP rapidly returned to baseline in phase 4, as expected given that the physiological indices normalize and the resistance resolves during this phase. In addition, autonomic activity can frequently influence IOP, the increased HR and LF/HF ratio found in phase 2 suggest sympathetic activation, while the significant increase in HF and the marked decrease in HR in phase 4 suggest parasympathetic excitation. These results contrast with previous studies showing that sympathetic nervous system activation can lead to a decrease in IOP, while parasympathetic over-activation can produce an elevation of IOP [23, 25, 26]. Our regression analysis also showed a significant correlation of HRV with IOP. We thought this result reflected the synchronism between the changes of IOP and autonomic nerve excitability during the VM. Thus, we speculate that the IOP fluctuation arises from changes in blood flow, and that ocular anatomy may counteract and reverse the influences of autonomic activity.

The SC is the vein at the chamber angle that collects aqueous humor from the anterior chamber and delivers it into the bloodstream [27]. Chen et al. found that SC collapse may be a cause of the IOP peak after the water-drinking test [28]. Numerous studies have also reported that an IOP of 30–50 mmHg can cause distention of the trabecular sheets in the SC, and reduce the size of the SC lumen [29, 30]. However, in the present study, the increase in SCAR from baseline to phase 2, with a concurrent increase in IOP, is harder to explain. As the SC was suggested have autonomic regulation functions [25, 31, 32], the expansion and collapse of the SC may not be completely dependent on the IOP. Speculatively, expansion of SC may be caused by sympathetic nerve stimulation in phase 2 of the VM. Although the average SCAR in phase 4 was reduced compared with baseline and phase 2, the changes were not significant. Activation of the parasympathetic nervous system was previously reported to be involved SC collapse, and parasympathetic excitation was also reported in phase 4 [25, 28]. However, we found only 13 individuals with a smaller SCAR in phase 4 than that at baseline. Speculatively, this may relate to individual differences in the rate of autonomic regulation. Thus, the recovery of normal breathing after 15 s of the VM in the present study may be too short for some individuals to finish the regulation.

MOPP represents the gradient of efficient perfusion for all intraocular structures, including the optic nerve head and the retina [33]. The marked elevation in IOP in phase 2 in the present study was associated with a reduction in MOPP, which started to increase in phase 4 and rapidly recovered. Interestingly, mechanical and ischemic damage to the optic nerve head was suggested to lead to the glaucoma process in phase 2 of the VM [33, 34].

The VM is widely used in daily life, and is done automatically and briefly [1]. Changes caused by the VM in healthy young individuals may carry no clinical significance, although for patients with high risk factors of glaucoma, we suggest to avoid repeating VMs in daily life.

This study has some limitations. First, subjects only performed the WM for 15 s before recovering normal breath, which may be too short for physiological indicators to resolve in all individuals. It also remains unclear whether similar effects of the VM are observed in elderly subjects or patients with glaucoma, as all of our individuals were young and healthy. Finally, we can’t measure the thickness of the anterior choroid in AS-OCT images. Thus, more detailed data sets are required in future studies, and mechanical and ischemic damage caused by the VM to the optic nerve head also needs further researches in the future.

## Conclusions

The expansion and collapse of the SC in different phases of the VM may be caused by changes in autonomic nervous system activity, while the effects of the VM on IOP may relate to changes in blood flow and ocular anatomy.

## Data Availability

The datasets used and/or analysed during the current study are available from the corresponding author on reasonable request.

## Abbreviations

AOD500: Angle opening distance at 500 μm from the scleral spur
ARA750: Angle recess area at 750 μm from the scleral spur
AS-OCT: Anterior optical coherence tomography
BCVA: Best corrected visual acuity
DBP: Diastolic blood pressure
ECGs: Electrocardiograms
HF: High frequency
HR: Heart rate
HRV: Heart rate variability
IOP: Intraocular pressure
LF/HF: The ratio of low frequency power and high frequency power
MAP: Mean arterial pressure
MOPP: Mean ocular perfusion pressure
PD: Pupil diameter
RE: Refractive error
SBP: Systolic blood pressure
SC: Schlemm’s canal
SCAR: SC area
TIA500: Trabecular iris angle at 500 μm from the scleral spur
TISA500: Trabecular-iris space area at 500 μm from the scleral spur
VM: Valsalva maneuver

## References

[1] Jellinek EH (2006). The Valsalva manoeuvre and Antonio Valsalva (1666-1723). J R Soc Med.

[2] Palamar M, Dag MY, Yagci A (2015). The effects of Valsalva manoeuvre on ocular response analyzer measurements. Clin Exp Optom.

[3] Albert BL, M. D. (1966). A simple test of cardiac function based upon the heart rate changes induced by the valsalva maneuver. Am J Cardiol.

[4] Benarroch EE, Opfer-Gehrkin TL (1991). Low. PA. Use of the photoplethysmographic technique to analyze the Valsalva maneuver in normal man. Muscle Nerve.

[5] Brody S, Erb C, Veit R, Rau H (1999). Intraocular pressure changes: the influence of psychological stress and the Valsalva maneuver. Biol Psychol.

[6] Goldstein DS, Cheshire WP (2017). Beat-to-beat blood pressure and heart rate responses to the Valsalva maneuver. Clin Auton Res.

[7] Ricci S, Moro L, Minotti GC, Incalzi RA, De Maeseneer M (2018). Valsalva maneuver in phlebologic practice. Phlebology.

[8] Pstras L, Thomaseth K, Waniewski J, Balzani I, Bellavere F (2016). The Valsalva manoeuvre: physiology and clinical examples. Acta Physiol (Oxf).

[9] Korner PI, Tonkin AM, Uther JB (1976). Reflex and mechanical circulatory effects of graded Valsalva maneuvers in normal man. J Appl Physiol.

[10] Stodtmeister R, Heyde M, Georgii S, Matthe E, Spoerl E, Pillunat LE (2018). Retinal venous pressure is higher than the airway pressure and the intraocular pressure during the Valsalva manoeuvre. Acta Ophthalmology.

[11] Kara N, Kenan S (2018). Effect of refractive status on Valsalva-induced anterior segment changes. Int Ophthalmol.

[12] Schuman JS, Massicotte EC, Connolly S, Hertzmark E, Mukherji B, Kunen MZ (2000). Increased intraocular pressure and visual field defects in high resistance wind instrument players. Ophthalmology.

[13] Raczynski JM, Mason DA, Wilson RP, Silvia ESM, Kleinstein RN (1985). Muscular and intraocular pressure responses among ocular-hypertensive subjects: is there a rationale for biofeedback?. Biofeedback Self-Regul.

[14] Yan X, Li M, Song Y, Guo J, Zhao Y, Chen W, Zhang H (2016). Influence of exercise on intraocular pressure, Schlemm's canal, and the trabecular meshwork. Invest Ophthalmol Vis Sci.

[15] Chen W, Chen L, Chen Z, Xiang Y, Liu S, Zhang H, Wang J (2018). Influence of the water-drinking test on intraocular pressure, Schlemm's canal, and autonomic nervous system activity. Invest Ophthalmol Vis Sci.

[16] Dada T, Gupta V, Deepak KK, Pandey RM (2006). Narrowing of the anterior chamber angle during Valsalva maneuver: a possible mechanism for angle closure. Eur J Ophthalmol.

[17] Gherghel D, Orgul S, Gugleta K, Gekkieva M, Flammer J (2000). Relationship between ocular perfusion pressure and retrobulbar blood flow in patients with glaucoma with progressive damage. Am J Ophthalmol.

[18] Leske MC (2003). Factors for Glaucoma progression and the effect of treatment. Arch Ophthalmol.

[19] Sztajzel J. Heart rate variability: a noninvasive electrocardiographic method to measure the autonomic nervous system. Swiss Med Wkly 2004, 134(35–36):514–522.; doi: 2004/35/smw-10321. 10.4414/smw.2004.10321 15517504

[20] McDougal DH, Gamlin PD (2015). Autonomic control of the eye. Compr Physiol.

[21] Henry BL, Minassian A, Paulus MP, Geyer MA, Perry W (2010). Heart rate variability in bipolar mania and schizophrenia. J Psychiatr Res.

[22] Kleiger RE, Bigger JT, Bosner MS, Chung MK, Cook JR, Rolnitzky LM, Steinman R, Fleiss JL (1991). Stability over time of variables measuring heart rate variability in normal subjects. Am J Cardiol.

[23] Li F, Gao K, Li X, Chen S, Huang W, Zhang X (2017). Anterior but not posterior choroid changed before and during Valsalva manoeuvre in healthy Chinese: a UBM and SS-OCT study. Br J Ophthalmol.

[24] Chen X, Yang R, Kuang D, Zhang L, Lv R, Huang X, Wu F, Lao G, Ou S (2017). Heart rate variability in patients with major depression disorder during a clinical autonomic test. Psychiatry Res.

[25] Cerman E, Eraslan M, Dericioglu V, Sahin O, Cekic O, Mahmutyazicioglu K (2014). Choroidal varix elevates macula following Valsalva manoeuvre. Br J Ophthalmol.

[26] Khan CJ (2002). Pulsatile ocular blood flow: the effect of the Valsalva manoeuvre in open angle and normal tension glaucoma: a case report and prospective study. Br J Ophthalmol.

[27] Lee W, Bae HW, Kim CY, Seong GJ (2017). The change of anterior segment parameters after cataract surgery in normal-tension glaucoma. Int J Ophthalmol.

[28] Feibel RM (2015). Sympathectomy for glaucoma: its rise and fall (1898–1910). Surv Ophthalmol.

[29] Gherezghiher T, Hey JA, Koss MC (1990). Parasympathetic nervous control of intraocular pressure. Exp Eye Res.

[30] Mansouri K, Shaarawy T (2015). Update on Schlemm's canal based procedures. Middle East Afr J Ophthalmol.

[31] Johnstone MA, Grant WG (1973). Pressure-dependent changes in structures of the aqueous outflow system of human and monkey eyes. Am J Ophthalmol.

[32] Hann CR, Vercnocke AJ, Bentley MD, Jorgensen SM, Fautsch MP (2014). Anatomic changes in Schlemm's canal and collector channels in normal and primary open-angle glaucoma eyes using low and high perfusion pressures. Invest Ophthalmol Vis Sci.

[33] Zhou EH, Krishnan R, Stamer WD, Perkumas KM, Rajendran K, Nabhan JF, Lu Q, Fredberg JJ, Johnson M (2012). Mechanical responsiveness of the endothelial cell of Schlemm's canal: scope, variability and its potential role in controlling aqueous humour outflow. J R Soc Interface.

[34] Tamm ER, Braunger BM, Fuchshofer R (2015). Intraocular pressure and the mechanisms involved in resistance of the aqueous humor flow in the trabecular meshwork outflow pathways. Prog Mol Biol Transl Sci.

